# Emotion word processing: does mood make a difference?

**DOI:** 10.3389/fpsyg.2015.01191

**Published:** 2015-08-24

**Authors:** Sara C. Sereno, Graham G. Scott, Bo Yao, Elske J. Thaden, Patrick J. O'Donnell

**Affiliations:** ^1^School of Psychology, University of GlasgowGlasgow, UK; ^2^Institute of Neuroscience and Psychology, University of GlasgowGlasgow, UK; ^3^Applied Psychology Research Group, School of Media, Culture and Society, University of the West of ScotlandPaisley, UK; ^4^School of Psychological Sciences, University of ManchesterManchester, UK

**Keywords:** emotion, mood induction, valence, arousal, word frequency, visual word recognition, lexical decision

## Abstract

Visual emotion word processing has been in the focus of recent psycholinguistic research. In general, emotion words provoke differential responses in comparison to neutral words. However, words are typically processed within a context rather than in isolation. For instance, how does one's inner emotional state influence the comprehension of emotion words? To address this question, the current study examined lexical decision responses to emotionally positive, negative, and neutral words as a function of induced mood as well as their word frequency. Mood was manipulated by exposing participants to different types of music. Participants were randomly assigned to one of three conditions—no music, positive music, and negative music. Participants' moods were assessed during the experiment to confirm the mood induction manipulation. Reaction time results confirmed prior demonstrations of an interaction between a word's emotionality and its frequency. Results also showed a significant interaction between participant mood and word emotionality. However, the pattern of results was not consistent with mood-congruency effects. Although positive and negative mood facilitated responses overall in comparison to the control group, neither positive nor negative mood appeared to additionally facilitate responses to mood-congruent words. Instead, the pattern of findings seemed to be the consequence of attentional effects arising from induced mood. Positive mood broadens attention to a global level, eliminating the category distinction of positive-negative valence but leaving the high-low arousal dimension intact. In contrast, negative mood narrows attention to a local level, enhancing within-category distinctions, in particular, for negative words, resulting in less effective facilitation.

## Introduction

For several decades, research into visual word recognition has sought to identify and delineate the factors affecting the access of word meaning. One focus of more recent research has been on the processing of written emotional words. In general, such research has established that emotion words provoke differential responses in comparison to neutral words. Words, however, are typically recognized not in isolation, but within a context. A context can be the prior sentence or paragraph that makes a word more or less predictable. Alternatively, a context can be the inner emotional state of the comprehender. The current study investigates the effect of induced mood on the recognition of emotional and neutral words. We begin by reviewing recent advances in emotion word recognition. We then consider studies that have investigated how mood affects word recognition. Our study attempts to address some of the perceived limitations of the research that has been conducted to date.

Emotion words are typically characterized within a two-dimensional framework of valence, a measure of value or worth, and arousal, a measure of internal activation (e.g., Osgood et al., 1957; Russell, 1980). Because extreme valence is correlated with higher arousal (e.g., Bradley and Lang, 1999), positive and negative words, when compared with neutral words, also tend to have higher associated levels of arousal. In terms of their semantics, emotion words, broadly construed, can either express an emotional state (e.g., *happy, panic*) or elicit one (e.g., *puppy, shark*).

Investigations of emotion word processing have often examined different categories of emotion words, controlled for different lexical variables, and used diverse experimental paradgims, making direct comparisons and generalizations difficult (Scott et al., 2009, 2012). Until more recently, most studies did not compare positive, negative, and neutral words within an experiment, but instead examine only two of these three categories or sometimes words comprising specific emotional categories (e.g., happiness, sadness). Nonetheless, a processing advantage for positive over neutral words is generally demonstrated (e.g., Kanske and Kotz, 2007; Kuchinke et al., 2007; Kousta et al., 2009; Schacht and Sommer, 2009; Scott et al., 2009, 2012, 2014; Sheikh and Titone, 2013; Knickerbocker et al., 2015). Some studies have shown an advantage for negative over neutral words (e.g., Tabert et al., 2001; Windmann et al., 2002; Nakic et al., 2006; Kanske and Kotz, 2007; Kousta et al., 2009; Schacht and Sommer, 2009; Knickerbocker et al., 2015). Others have shown an advantage for positive over negative words (e.g., Kiehl et al., 1999; Wentura et al., 2000; Dahl, 2001; Atchley et al., 2003; Estes and Adelman, 2008; Citron et al., 2014; Kuperman et al., 2014).

Research of ours and of others has investigated the interaction of emotion with word frequency (Kuchinke et al., 2007; Scott et al., 2009, 2012, 2014; Méndez-Bértolo et al., 2011; Sheikh and Titone, 2013). A word frequency effect represents the behavioral advantage in recognizing commonly used high frequency (HF) words over low frequency (LF) words that occur less often (e.g., Hand et al., 2010, 2012). A word frequency effect is considered to be a reliable indicator of lexical access (e.g., Sereno and Rayner, 2003). Consequently, an interaction between word emotionality and frequency would imply that a word's emotional quality can influence the early, lexical stages of word recognition. Scott et al. (2009, 2012, 2014) have found such an interaction in lexical decision reaction times (RTs), in brain electrophysiology measures, and in eye fixation durations during fluent reading. The pattern of behavioral effects is as follows: for LF words, positive and negative word responses are faster than neutral word responses; for HF words, positive word responses alone are faster than negative or neutral word responses (which do not differ from each other). The differential pattern of responses to negative words across frequency may be able to account for the different patterns of emotion word effects in the literature in that different studies may have used different ratios of higher and lower frequency negative words within their stimulus sets. Nevertheless, converging evidence from recent brain electrophysiological studies has confirmed an early, lexical (i.e., before ~250 ms) locus of emotion in word recognition tasks (Herbert et al., 2006, 2008; Kissler et al., 2007, 2009; Scott et al., 2009; Bayer et al., 2012; Kissler and Herbert, 2013; Keuper et al., 2014; Zhang et al., 2014).

Another factor that influences the recognition of emotion words is the mood state of the reader. According to Bower's (1981) notion of mood congruency, there is a link between mood state and cognitive processes such as attention and memory, whereby processing is facilitated when the affective tone of received information matches the valence of the mood. A mood can be reliably induced in individuals via several different laboratory procedures (Martin, 1990), including the self-statement or Velten (1968) technique, music listening (Västfjäll, 2002), film watching, and hypnotic suggestion. For word recognition experiments, inducing mood via the non-verbal method of listening to instrumental music is generally preferred.

A number of studies have investigated mood effects on the recognition of emotion words (e.g., Small, 1985; Halberstadt et al., 1995; Niedenthal et al., 1997; Olafson and Ferraro, 2001; Ferraro et al., 2003). In these studies, a mood is first induced in participants by having them listen to either “happy” or “sad” music, and this is followed by a word recognition task (sometimes the music is also played in the background during the task). In general, these studies find that mood-congruent words are facilitated relative to mood-incongruent words. However, there are certain methodological concerns which may weaken the generalizability of the findings. We focus on the three studies that used lexical decision as the response time measure (Niedenthal et al., 1997; Olafson and Ferraro, 2001; Ferraro et al., 2003). In Halberstadt et al. (1995), participants wrote down auditorily presented words that were purposely selected as homophones having both emotional and non-emotional realizations (e.g., *won, one*). In Small (1985), words were presented tachistoscopically for increasing durations until they were identified.

Our concerns with the lexical decision studies were as follows. First, relatively few stimuli were used and lexical specifications of the stimuli were not always controlled or presented. Niedenthal et al.'s (1997) Experiments 1 and 2 used either six or eight words, respectively, within each of their four conditions (“happy words,” “sad words,” “love words,” and “anger words”) and equal numbers of neutral words (24 or 32, respectively). Olafson and Ferraro (2001) used 25 “happy words” and 25 “sad words” (which included homophones from Halberstadt et al., 1995); no neutral words were included. Ferraro et al. (2003) replicated Olafson and Ferraro (2001) with identical stimuli, extending the original experiment by testing older adults (N.B., the stimuli are not listed in either study). In fact, neither of these studies presented any lexical characteristics of their stimuli (e.g., frequency, length, valence, arousal). In Niedenthal et al. (1997), the stimuli were not explicitly controlled for arousal—happy words had numerically higher arousal values than the sad words (accd. to the norms of Bradley and Lang, 1999). In terms of mood induction, Niedenthal et al.'s (1997) Experiment 2 was the only study to include a control group of participants that were not exposed to any mood-inducing music. The selection of music chosen to induce the different moods is another concern. Across all studies, many of the happy and sad music pieces used are relatively well-known (e.g., Mozart's “Eine Kleine Nacht Musik,” and Barber's “Adagio for Strings”). As such, individuals' own affective associations may or may not be consistent with the desired mood that was to be induced. In addition, the tempo of the sad music is much slower than that of the happy music, which should correspondingly affect RTs (e.g., Kämpfe et al., 2010; Bottiroli et al., 2014). In all three studies, only the discrete emotions of happy and sad were examined [although it may be that Olafson and Ferraro's (2001) happy and sad words could be classified more generally as positive and negative words]. It is possible that implementing the broader positive and negative categories, derived from the dimensions of valence and arousal, may also demonstrate facilitation within a mood-induction framework (e.g., Eerola and Vuoskoski, 2011).

The current study attempted to address these concerns. As in our prior research (Scott et al., 2009, 2012, 2014), we implemented an Emotion (Positive, Negative, Netural) × Frequency (LF, HF) design. We used a total of 240 words, with 40 words in each of the 6 conditions. Words were matched across conditions on an item-by-item basis for word frequency and length. We also used several sets of published norms to obtain values on all our stimuli for valence and arousal, as well as imageability and age of acquisition (AoA). We induced positive and negative mood and also had a control condition in which no mood was induced. Positive and negative music clips were selected from a variety of sources that we anticipated would make them less recognizable (e.g., from movie soundtracks), with the deliberate selection of positive and negative clips having similar tempos. Music clips were normed ahead of time to ensure that they were equally intense in valence and arousal. Finally, we sought to broaden the scope of both the induced mood and emotional stimuli from discrete to categorical emotions (i.e., from “happy” and “sad” to “positive” and “negative”).

## Methods

### Participants

A total of 144 members of the University of Glasgow community participated in this study. All were native English speaking, had not been diagnosed with dyslexia, had normal or corrected-to-normal vision, had normal hearing, and were naïve as to the purpose of the experiment. An additional nine participants took part in the study, but their data were excluded due to a high amount of data loss from word errors, non-word errors, and/or very slow responses. Participants were compensated for their time with either experimental credits or £5. All participants gave written informed consent and the experimental procedure was approved by the College of Science and Engineering Ethics Committee at the University of Glasgow.

Participants were opportunistically assigned to one of the three mood groups—Control, Positive, and Negative. All groups comprised 48 participants. The average age and number of females within each group were as follows: 22 years and 35 females for the Control group; 23 years and 31 females for the Positive group; and 24 years and 39 females for the Negative group.

### Design and materials

A 3 (Mood: Control, Positive, Negative) × 3 (Emotion: Positive, Negative, Neutral) × 2 (Frequency: LF, HF) mixed design was used. Mood was the between-participants factor and was implemented via a mood-induction procedure for Positive and Negative groups (no mood induction was used for the Control group). Emotion and Frequency were within-participant factors and the different levels of these factors were achieved via stimulus selection based on existing norms and databases.

#### Mood induction stimuli

In accordance with previous studies, pieces of music were used to induce positive or negative mood (e.g., Eerola et al., 2009; Eerola and Vuoskoski, 2011). In order to select the appropriate music, a norming study was run on a set of 28 participants (mean age 20 years; 19 females), none of whom (later) took part in the main experiment. In view of constraints of the main experiment, it was necessary to have a large selection of musical pieces to contribute to the mood induction procedures. The participants were run in small groups in sessions lasting ~1.5 h. They were presented with 52 music clips, each lasting around 1 min. Participants were asked to rate each clip in terms of its valence and arousal, both on 9-point scales. Valence ranged from 1 (low, negative) to 9 (high, positive) and arousal ranged from 1 (low) to 9 (high). For each clip, participants were also asked to indicate whether they recognized it.

Based on the average valence and arousal ratings, individual pieces were then chosen for inclusion in the main experiment for mood induction. Positive music selections had valence ratings greater than 6 and negative music selections had valence ratings less than 4. Both positive and negative music selections had comparable arousal ratings of around 6. Since the main experiment included a large number of trials, we wanted to ensure that participants' moods were maintained throughout the experiment. Thus, we opted for three separate musical mood-induction exposures, each lasting around 5 min. Each 5-min set of music comprised five different pieces, with a total of 15 pieces for each mood induced. For these pieces (15 positive, 15 negative), participants' recognition rate was 19%. Thus, on average, participants reported recognizing just under three of the 15 pieces for each mood set. A complete list of the selected music is presented in Appendix A. The valence and arousal ratings (with SDs) from the final sets of positive and negative music are presented in Table 1.

**Table 1:** **Means (with SDs) of music specifications for Positive and Negative mood conditions**.

**Music**	**Set**	**Duration**	**Valence**	**Arousal**
**Positive**	**1**	319	7.6 (1.1)	6.0 (1.6)
	**2**	314	7.4 (1.2)	6.0 (1.5)
	**3**	299	7.5 (1.1)	5.8 (1.7)
**Negative**	**1**	317	2.7 (1.3)	6.0 (2.0)
	**2**	306	2.6 (1.2)	6.1 (1.7)
	**3**	298	2.7 (1.4)	5.9 (1.9)

*Units of measurement are as follows: Duration in seconds; Valence on a scale from 1 (low, negative) to 9 (high, positive); Arousal on a scale from 1 (low) to 9 (high)*.

#### Lexical decision stimuli

The 3 (Emotion: Positive, Negative, Neutral) × 2 (Frequency: LF, HF) design gave rise to 6 conditions. With 40 words in each of the 6 conditions, the lexical decision experiment comprised a total of 240 words, ranging from 3 to 9 characters in length. Non-words comprised 240 pronounceable, orthographically legal pseudowords that were matched to word stimuli in terms of string length (e.g., *wid, felp, chire, narvey, bruddle, durledge, slamperic*). Words were matched across the 6 conditions on an item-by-item basis for word frequency (occurrences per million) and word length (number of letters). The complete list of 240 words is presented in Appendix B. The specifications of the words in terms of length, frequency, valence, and arousal are presented in Table 2. Other word characteristics that were not directly controlled for, but were matched as best as possible across conditions, are also presented in Table 2. These include number of syllables, imageability (i.e., whether a word represents something that is easy or difficult to imagine or picture), age of acquisition (AoA; i.e., the age at which a word was initially learned), and grammatical class.

**Table 2:** **Means (with SDs) of target specifications across experimental conditions**.

**Variable**	**LF**			**HF**		
	**Positive**	**Negative**	**Neutral**	**Positive**	**Negative**	**Neutral**
**Length**	5.8 (1.5)	5.7 (1.4)	5.6 (1.3)	5.8 (1.5)	5.7 (1.6)	5.8 (1.2)
**Frequency**	9.0 (4.5)	9.4 (5.0)	9.3 (4.8)	65.8 (51)	63.3 (54)	62.6 (47)
**Valence**	7.6 (0.5)	2.4 (0.4)	5.1 (0.6)	7.8 (0.5)	2.4 (0.6)	5.3 (0.4)
**Arousal**	5.9 (0.8)	6.1 (0.7)	4.1 (0.7)	6.1 (0.7)	6.3 (0.8)	4.0 (0.5)
**Syllables**	1.9 (0.8)	1.8 (0.6)	1.7 (0.6)	1.8 (0.7)	1.8 (0.7)	1.9 (0.6)
**Imageability**	4.9 (1.1)	4.7 (1.0)	5.0 (1.2)	5.0 (0.9)	4.7 (0.8)	4.9 (1.2)
**AoA**	3.9 (1.2)	3.9 (0.9)	3.5 (0.9)	3.3 (0.8)	3.7 (0.9)	3.3 (0.9)
**PoS**						
** Adjective**	0.25	0.28	0.23	0.35	0.25	0.13
** Noun**	0.73	0.63	0.73	0.78	0.70	0.93
** Verb**	0.33	0.43	0.20	0.18	0.43	0.20

*LF, low frequency; HF, high frequency; AoA, age of acquisition; PoS, part of speech. Units of measurement are as follows: Length in number of letters; Frequency in occurrences per million; Arousal on a scale from 1 (low) to 9 (high); Valence on a scale from 1 (low, negative) to 9 (high, positive); Syllables in number of syllables; Imageability on a scale from 1 (low) to 7 (high); AoA on a scale from 1 (early) to 7 (late). For PoS, the grammatical class of each word was determined (some words were classified as belonging to more than one class), and the average frequencies of Adjective, Noun, and Verb usage across conditions are listed*.

The different types of emotion words were determined by their valence values from the Affective Norms for English Words (ANEW), a database of 1000 words (Bradley and Lang, 1999). Each word has associated ratings for valence, from 1 (low, having a negative meaning) to 9 (high, having a positive meaning), and for arousal, from 1 (low) to 9 (high). As extreme valence values correlate with higher levels of arousal (Bradley and Lang, 1999), Positive and Negative words also tended to have higher arousal ratings. Mean valence and arousal values (with SDs) across all word conditions are presented in Table 2.

Word frequencies were obtained from the British National Corpus (BNC; http://www.natcorp.ox.ac.uk), a corpus of 90 million written-word tokens, using the on-line resource provided by Davies (2004; http://corpus.byu.edu/bnc). Word frequencies (with SDs) across all conditions are presented in Table 2.

While the chief variables affecting the speed of recognizing a word are its length, frequency, and contextual predictability, several other lexical variables are also known to influence processing of words (e.g., Sereno et al., 2009; Yao et al., 2013). For example, high imageable or early AoA words are facilitated relative to low imageable or late AoA words (e.g., Juhasz and Rayner, 2003; Balota et al., 2004; Sereno and O'Donnell, 2009). In addition, the grammatical class of a word also affects its processing (e.g., Sereno, 1999; Palazova et al., 2011). Means (with SDs) of these variables across all conditions are presented in Table 2. Imageability ratings were obtained from five sources: the Bristol Norms (Stadthagen-Gonzalez and Davis, 2006), the MRC Psycholinguistic Database (Wilson, 1988), and norms of Bird et al. (2001), Clark and Paivio (2004), and Cortese and Fugett (2004). AoA ratings were obtained from the first four sources listed for imageability as well as the norms of Morrison et al. (1997).

### Apparatus

Stimuli were presented via E-Prime 2.0 software (Psychology Software Tools, Pittsburgh, PA) on a Dell OptiPlex GX520 desktop computer and a 17″ LCD flat-screen monitor (1024 × 768 resolution; 75 Hz). Letter strings appeared in Courier New, 24-point bold font (black characters on a white background). At a viewing distance of ~84 cm, 2.3 characters of text subtended 1° of visual angle. Responses were made via the computer keyboard and were recorded with millisecond accuracy. Music was played through headphones and was adjusted to a comfortable volume.

### Procedure

Participants were tested individually. They were given written information about the experiment and a consent form. Participants were assigned (in order of their arrival) to one of the three mood groups. Participants were given mood assessment sheets to rate the current state of their mood via the dimensions of valence and arousal (described below). Participants in all groups rated their mood at the beginning of the experiment. They were then given instructions for the lexical decision task. They were told that half of the stimuli were words and half were non-words and that they should respond as quickly and as accurately as possible. They were instructed to make word responses using their right forefinger on the “L” key (labeled “W”) and non-word responses with their left forefinger on the “S” key (labeled “NW”). They were then presented with a short practice block of items (*N* = 12) to become accustomed to the task.

Each trial consisted of the following events. A blank screen was initially presented for 1000 ms. A fixation cross (+) then appeared in the center of the screen for 200 ms, replaced by another blank screen for 500 ms. A letter string was then presented centrally until the participant responded. Experimental trials (240 words, 240 non-words) were presented in a different random order for each participant.

The lexical decision experiment was presented in three equal blocks of trials. Participants in the Control mood condition performed the experiment with short break periods preceding each block. The procedure for participants in the Positive and Negative mood conditions was as follows. For each of the three blocks, they first listened to a set of mood-appropriate music (~5 min), rated their mood, then proceeded with a block of lexical decision trials. Positive and Negative mood condition participants were not asked whether they recognized any of the music (a total of 15 clips over the course of the experiment). We thought this would disrupt the flow of the experiment. Moreover, as these participants were selected from the same participant pool as those who had provided ratings for the pieces (none were the same), we assumed that recognition rates would be similarly minimal. The experiment lasted ~30 min for the Control mood participants and 45 min for Positive and Negative mood participants.

The mood rating sheets provided the following information to participants. Valence was described as a measure of value or worth and used a 9-point scale from 1 (very negative) to 5 (neutral) to 9 (very positive). Scale endpoints of “very positive” and “very negative” would indicate that they felt very good and very bad, respectively. Arousal was described as a measure of excitement vs. calmness and used a 9-point scale from 1 (very low arousal) to 5 (intermediate arousal) to 9 (very high arousal). The scale endpoint of “very high arousal” would indicate that they felt stimulated, excited, frenzied, jittery, or wide-awake, and that of “very low arousal” would indicate feeling relaxed, calm, sluggish, dull, or sleepy.

## Results

### Mood induction manipulation check

At the outset of the experiment (prior to any mood induction procedure), all participants provided valence and arousal ratings of their current mood. Mean ratings (with SDs) across the participant groups are presented in Table 3. A 1-factor analysis of variance (ANOVA) was carried out on the valence and arousal rating data comparing the three mood groups. No differences in ratings between mood groups were found either for valence [*F*_1(2, 141)_ = 1.09, *p* > 0.30] or for arousal [*F*_1_ < 1].

**Table 3:** **Means (with SDs) of valence and arousal ratings across mood groups during the experiment**.

**Measure**	**Control Mood**	**Positive Mood**	**Negative Mood**
**BEFORE EXPERIMENT**			
**Valence**	6.0 (1.4)	6.2 (1.4)	6.4 (1.4)
**Arousal**	4.6 (1.8)	4.9 (1.7)	5.0 (1.7)
**AFTER MUSIC**			
**Valence**	N/A	6.6 (1.2)	5.0 (1.3)
**Arousal**	N/A	5.4 (1.4)	5.8 (1.6)

*Units of measurement are as follows: Valence on a scale from 1 (low, negative) to 9 (high, positive); Arousal on a scale from 1 (low) to 9 (high)*.

For the Positive and Negative mood groups, participants listened to positive and negative music, respectively, before each of the three blocks of lexical decision trials. Participants in these mood groups provided additional ratings of their mood on each of these occasions. Mean valence and arousal ratings (with SDs) for Positive and Negative mood groups are presented in Table 3. Paired-sample *t*-tests were carried out separately for Positive and Negative mood groups, comparing their pre-experiment to post-music valence and arousal mood ratings. The Positive mood group showed a significant increase in valence (+0.4) [*t*_(47)_= 2.46, *p* < 0.05], and a marginal increase in arousal (+0.5) [*t*_(47)_ = 1.97, *p* = 0.055]. The Negative mood group showed a significant decrease in valence (−1.4) [*t*_(47)_ = −7.44, *p* < 0.001], as well as a significant increase in arousal (+0.8) [*t*_(47)_ = 3.36, *p* < 0.01].

### Lexical decision data

For correct word responses (97.77% of the data), items having RTs less than 250 ms or greater than 1500 ms were first excluded. In addition, for each participant in each condition, items with RTs beyond two standard deviations of the mean were then excluded. These trimming procedures resulted in an average data loss of 5.78% per participant (~2 items per condition). Overall, participants on average provided RT data on 37 of the 40 possible items per condition.

**Figure 1:**
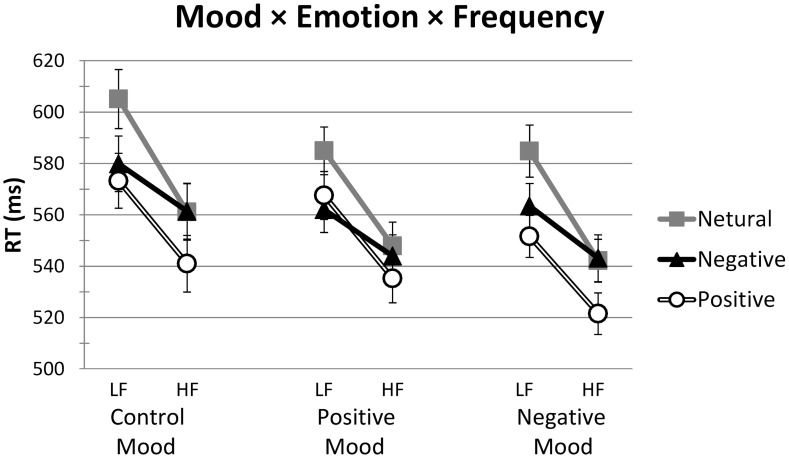
**Mean RT (ms), with SE bars, on words as a function of Mood (Control, Positive, Negative), Emotion (Positive, Negative, Neutral), and Frequency (LF, HF)**. LF, low frequency; HF, high frequency.

**Figure 2:**
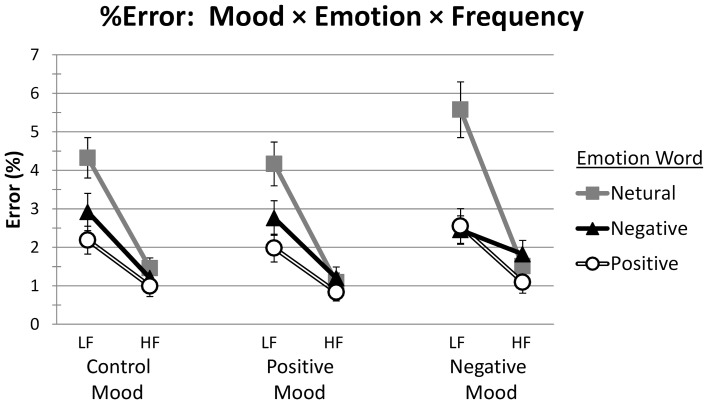
**Mean %Error, with SE bars, on words as a function of Mood (Control, Positive, Negative), Emotion (Positive, Negative, Neutral), and Frequency (LF, HF)**. LF, low frequency; HF, high frequency.

The mean RT data across experimental conditions are presented in Table 4. The RT means (with standard error bars) are presented in Figure 1. A three-way mixed design ANOVA was performed on the RT data both by participants (*F*_1_) and by items (*F*_2_). Mood (Control, Positive, Negative) was the between-participant factor; within-participant factors were the word variables of Emotion (Positive, Negative, Neutral) and Frequency (LF, HF). A summary of all RT main effects and interactions is presented in Table 5. The mean percent error (%Error) data are also presented and similarly analyzed (see Tables 4, 5, and Figure 2). However, as errors only comprised 2.23% of the total data, our focus is on the RT data.

**Table 4:** **RT and %Error means (with SDs) as a function of Mood (Control, Positive, Negative), Emotion (Positive, Negative, Neutral), and Frequency (LF, HF)**.

**Mood**	**Frequency**	**RT**				**%Error**		
		**Positive**	**Negative**	**Neutral**		**Positive**	**Negative**	**Neutral**
**Control**	**LF**	573 (74)	580 (75)	585 (70)		2.19 (2.5)	2.92 (3.4)	4.32 (3.6)
	**HF**	541 (76)	561 (75)	561 (76)		0.99 (2.0)	1.20 (1.9)	1.46 (1.8)
**Positive**	**LF**	568 (65)	562 (62)	585 (64)		1.98 (2.5)	2.76 (3.1)	4.17 (3.9)
	**HF**	535 (66)	544 (57)	548 (64)		0.83 (1.6)	1.20 (2.0)	1.09 (1.6)
**Negative**	**LF**	552 (57)	563 (61)	585 (70)		2.55 (3.1)	2.45 (2.6)	5.57 (5.0)
	**HF**	522 (56)	543 (63)	542 (58)		1.09 (2.0)	1.83 (2.5)	1.51 (2.0)

*RT in ms; LF, low frequency; HF, high frequency*.

**Table 5:** **Main effects and interactions by participants (*F*_1_) and by items (*F*_2_) for RT and %Error measures**.

**Source**	***df***	**RT**			**%Error**		
		***F***	***MSE***	***p***	***F***	***MSE***	***p***
**MOOD**							
*F*_1_	2141	1.13	24,993	>0.30	< 1		
*F*_2_	2117	17.70	1292	< 0.001	1.42	11	>0.20
**EMOTION**							
*F*_1_	2282	102.78	360	< 0.001	29.68	5	< 0.001
*F*_2_	2234	69.81	491	< 0.001	16.33	8	< 0.001
**FREQUENCY**							
*F*_1_	1141	374.89	539	< 0.001	118.83	7	< 0.001
*F*_2_	1117	249.33	720	< 0.001	69.69	10	< 0.001
**EMOTION** × **FREQUENCY**							
*F*_1_	2282	26.84	334	< 0.001	21.73	5	< 0.001
*F*_2_	2234	20.46	415	< 0.001	9.04	9	< 0.001
**MOOD** × **EMOTION**							
*F*_1_	4282	4.88	360	< 0.001	< 1		
*F*_2_	4234	2.97	491	< 0.05	< 1		
**MOOD** × **FREQUENCY**							
*F*_1_	2141	< 1			< 1		
*F*_2_	2117	< 1			< 1		
**MOOD** × **EMOTION** × **FREQUENCY**							
*F*_1_	4282	< 1			2.00	5	=0.095
*F*_2_	4234	< 1			< 1		

*MSE, mean squared error*.

#### RTs

##### Main effects

The between group factor of Mood was not significant by participants, but was significant by items (see Table 5). This disparity resulted from the much higher level of variance among participants than items (evidenced in the *MSE*s). Unlike participants, items were matched across groups. Bonferroni pairwise comparisons in the items analysis showed that participants in the Control mood condition (571 ms) were slower than those in both the Positive (557 ms) and Negative (552 ms) mood conditions [*p*_2_s < 0.001], which did not differ from each other [*p*_2_s > 0.30].

The main effect of Emotion was significant (see Table 5). Bonferroni pairwise comparisons by participants and items demonstrated reliable differences between all word types, with Positive words (548 ms) responded to faster than both Negative (559 ms) and Neutral (571 ms) words, which also significantly differed from each other [all *p*s < 0.001].

The main effect of Frequency was also significant (see Table 5). Responses to HF words (544 ms) were faster than those to LF words (575 ms).

##### Interactions

**Figure 3:**
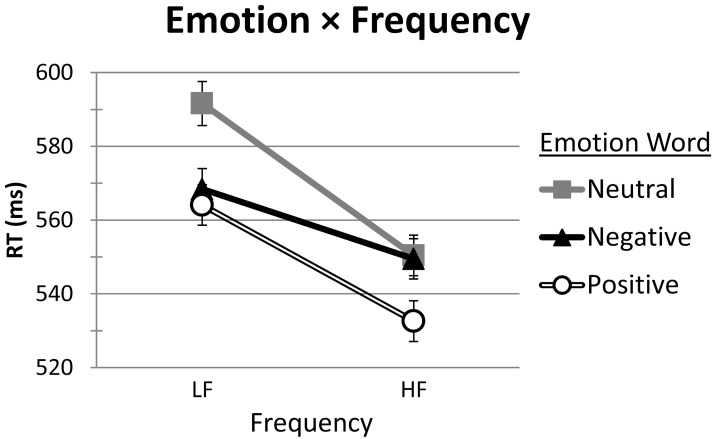
**Mean RT (ms), with SE bars, on words as a function of Emotion (Positive, Negative, Neutral) and Frequency (LF, HF)**. LF, low frequency; HF, high frequency.

Two of the interactions were significant: Emotion × Frequency and Mood × Emotion (see Table 5). The associated RT means (with standard error bars) for these interactions are presented in Figures 2, 3, respectively. Neither the Mood × Frequency nor the Mood × Emotion × Frequency interactions were significant (see Table 5).

For the Emotion × Frequency interaction (see Figure 3), participant and item Bonferroni pairwise comparisons examined frequency effects for each type of emotion word and emotion word differences within each level of frequency. Word frequency effects were significant for all types of emotion words [all *p*s < 0.001]. RTs to HF Positive, Negative, and Neutral words (533, 550, and 550 ms, respectively) were faster than those to their LF counterparts (564, 568, and 592 ms, respectively). For LF words, RTs to Positive (564 ms) and Negative (568 ms) words were faster than those to Neutral words (592 ms) [*p*s < 0.001]. The LF Positive-Negative contrast was marginal by participants [*p*_1_ = 0.099], and not significant by items [*p*_2_ > 0.25]. For HF words, a different pattern emerged. RTs to HF Positive words (533 ms) were significantly faster than those to both HF Negative (550 ms) and Neutral (550 ms) words [all *p*s < 0.001], which did not differ from each other [all *p*s = 1].

**Figure 4:**
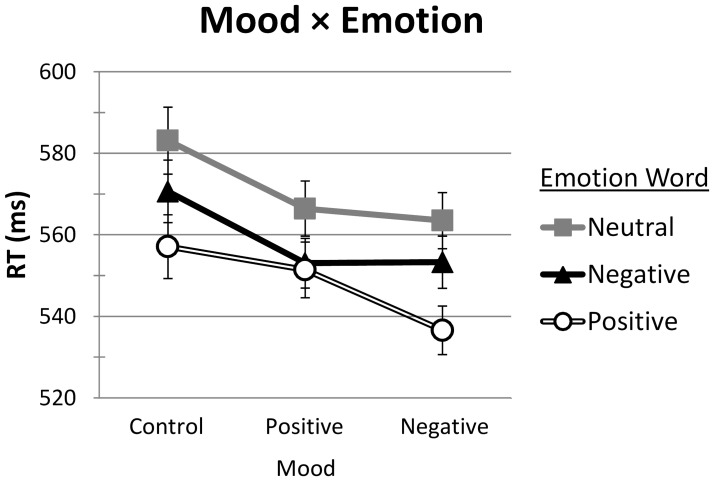
**Mean RT (ms), with SE bars, on words as a function of Mood (Control, Positive, Negative) and Emotion (Positive, Negative, Neutral)**.

For the Mood × Emotion interaction (see Figure 4), participant and item Bonferroni pairwise comparisons examined mood effects for each type of emotion word as well as emotion word differences within each level of mood. By participants, Control, Positive, and Negative mood groups did not differ significantly in their responses to Positive words (557, 551, and 536 ms, respectively), Negative words (571, 553, and 553 ms, respectively), nor Neutral words (583, 566, and 563 ms, respectively) [all *p*_1_s > 0.35]. The lack of significance (given apparent differences) is due to the high variability in RTs across participants. Item variability, in contrast, is much less as items are matched across groups (cf. the main effect of Mood). By items, significant differences did emerge. The Control mood group was significantly slower than the Positive and Negative mood groups in response to Negative words (571 ms vs. 553 and 553 ms, respectively) [*p*_2_s < 0.001] and to Neutral words (583 ms vs. 566 and 563 ms, respectively) [*p*_2_s < 0.01]. Positive and Negative mood groups did not differ in response to either Negative or Neutral words [*p*_2_s = 1]. In partial contrast, both the Control and Positive mood groups were significantly slower than the Negative mood group in response to Positive words (557 and 551 ms vs. 536 ms, respectively) [*p*_2_s < 0.001]. The difference between Control and Positive mood groups to Positive words was not significant [*p*_2_ > 0.15].

Participant and item Bonferroni pairwise comparisons also examined emotion word differences within each level of mood. Within the Control mood group, Positive words (557 ms) were responded to faster than Negative words (571 ms), and both types of words were responded to faster than Neutral words (583 ms) [all *p*s < 0.001]. A similar pattern emerged for the Negative mood group: Positive words (537 ms) were responded to faster than Negative words (553 ms), and both types of words were responded to faster than Neutral words (563 ms) [all *p*s < 0.01]. Within the Positive mood group, however, there was no difference between Positive (551 ms) and Negative (553 ms) words [all *p*s = 1], although both types of emotion words were responded to faster than Neutral words (566 ms) [all *p*s < 0.001].

#### %Error

##### Main effects

The between group effect of Mood was not significant (see Table 5). Similar to the RT findings, the within-participant effects of Emotion and Frequency were both significant (see Table 5). For Emotion, Bonferroni pairwise comparisons by participants and items demonstrated that more errors were reliably made with Neutral (3.02%) compared to both Positive (1.61%) and Negative (2.06%) words [all *p*s < 0.001]. Errors to Positive and Negative words differed significantly by participants [*p*_1_ < 0.05], but marginally by items [*p*_2_ = 0.071]. For Frequency, participants made fewer errors on HF (1.24%) than on LF (3.21%) words.

##### Interactions

The only interaction that was significant was Emotion × Frequency (see Table 5). Participant and item Bonferroni pairwise comparisons examined frequency effects for each type of emotion word and emotion word differences within each level of frequency. Word frequency effects were significant for all types of emotion words [all *p*s < 0.001]. The percentage of errors on HF Positive, Negative, and Neutral words (0.97, 1.41, and 1.35%, respectively) was less than that on their LF counterparts (2.24, 2.71, and 4.69%, respectively). For LF words, significantly fewer errors were made on both Positive (2.24%) and Negative (2.71%) words in comparison to Neutral words (4.69%) [all *p*s < 0.001]. There was no difference between errors on Positive and Negative words [all *p*s > 0.20]. For HF words, none of the comparisons reached significance. The %Error on Positive words (0.97%) was marginally less than that on Negative words (1.41%) [*p*_1_ = 0.062, *p*_2_ = 0.086], and no different than that on Neutral words (1.35%) [all *p*s > 0.10]. Negative and Neutral words did not differ in %Error [all *p*s = 1].

## Discussion

The current study investigated effects of mood on emotion word recognition. While past studies have demonstrated mood-congruency effects (e.g., Niedenthal et al., 1997; Olafson and Ferraro, 2001; Ferraro et al., 2003), they may be limited by the methodologies that were employed. For example, tight experimental control over lexical variables associated with the stimuli was not always implemented, baseline conditions (i.e., neutral words, no mood induction) were not always used, happy and sad mood-inducing music differed in tempo and arousal, and effects were restricted to discrete emotions (i.e., happy, sad). We attempted to address these concerns. In our study, our between-group factor of mood was induced via positive and negative music equated for intensity of valence and arousal. A no-mood control group was also included. In line with recent emotion word studies, we used an Emotion (Positive, Negative Neutral) × Frequency (LF, HF) stimulus design. Word stimuli (*N* = 240) varied systematically in valence and arousal and were explicitly controlled for word frequency and length. In contrast to the prior mood-induction studies, we also attempted to match stimuli as closely as possible for imageability, AoA, and grammatical class, although strict equivalences of these variables were not always achieved (see Table 2) which could limit the generalizability of our findings.

We found main effects of Mood (significant only by items due to inter-participant variability), Emotion, and Frequency. Positive and Negative mood groups were faster overall in their responses than the Control (no music) group. This was most likely due to participants' relatively higher levels of arousal produced by the mood-inducing music (see Table 3) as well as a possible consequence of the music's tempo (e.g., Husain et al., 2002; Kämpfe et al., 2010; Bottiroli et al., 2014). The Emotion-Frequency results are similar to what we have found in the past (Scott et al., 2009, 2012, 2014). For Emotion, Positive words were responded to faster than Negative words, and both had faster responses than Neutral words. For Frequency, HF words were responded to faster than LF words. The Emotion × Frequency interaction arose from the pattern associated with Negative words—responses to LF Negative words were as fast as Positive words (both faster than Neutral words), whereas responses to HF Negative words were as slow as Neutral words (both slower than Positive words). The relative slowing of responses to negative (vs. positive) stimuli has often been explained by differential effects at different stages of stimulus processing. Two-stage models of emotion word processing—Taylor's (1991) mobilization-minimization hypothesis and Pratto and John's (1991) automatic vigilance hypothesis—propose that all emotionally valenced words enjoy an initial facilitation relative to neutral words because of their high arousal, but that negative words are subsequently inhibited due to their low valence and, hence, inherent threat. This would predict a consistent advantage in processing for positive over neutral words, and an advantage for negative over neutral words under some circumstances. Scott et al. (2009) suggested that salience in the form of word frequency may be one such moderating factor. Various models of this process have been reviewed by Kuperman (2015) who distinguished between the “motivated attention” account, explaining equal speeding of positive and negative words, and the “automatic vigilance” account, which argues for fast attention capture in negative words but slower disengagement, producing a relative advantage for positive over negative words.

The main aim of our study, however, was to investigate the effect of mood on the processing of emotion words. We had expected to find mood-congruency effects within the more general categories of “positive” and “negative.” Although we found a significant Mood × Emotion interaction, it did not appear to be the result of mood-congruency effects (see Figure 4). Instead, we found that Neutral and Negative mood conditions behaved similarly, mirroring the main effect of Emotion (with fastest responses to Positive words, followed by Negative, then Neutral words). In the Positive mood condition, the relative advantage for Positive words disappeared— responses to Positive and Negative words did not differ, but both were faster than responses to Neutral words. From these findings, we are left with two patterns of data to explain. First, for the Positive mood group, mood congruency would predict that responses to Positive words should be even faster than that found in a baseline (Control mood) condition. In fact, there was no difference between responses to Positive and Negative words. It is not clear whether this represents a relative slowing down of responses to Positive words or a relative speeding up of responses to Negative words. Second, for the Negative mood group, mood congruency would again predict that responses to Negative words should be speeded in comparison to the Control mood condition. On this view, responses to Negative words should be as fast or faster than those to Positive words. However, our results showed that the Negative mood group behaved no differently than the Control group, with the exception that the overall response time was speeded.

It has been proposed that internal affective cues can direct our attention, with positive mood focusing attention on the metaphorical forest and negative on the trees (e.g., Easterbrook, 1959; Gasper and Clore, 2002; Fredrickson and Branigan, 2005; Huntsinger, 2013). This is traditionally attributed to a broadening or narrowing of attention to the global or local level, respectively. Within this context, it becomes possible to account for the pattern of our findings. For the Positive mood group, a broadening of attention could diminish the impact of any negative content of words in the second stage of a two-stage processing mechanism, removing the need to inhibit the processing of negative stimuli and eliminating the difference in response time between positive and negative emotional stimuli. A positive mood might act as a buffer against potential threat inherent in negative stimuli (e.g., Das et al., 2012). Under such circumstances, the initial processing advantage (or “mobilization”) enjoyed by negative words would not only be maintained, but would be preserved because the subsequent inhibition (or “minimization”) stage would not be prompted. In this way, positive mood could eliminate the category distinction of positive-negative valence but leave the high-low arousal dimension intact. For the Negative mood group, a narrowing of attention could enhance distinctions between words within each of the categories of Positive, Negative, and Neutral. Traditionally, emotions have been classified into six subtypes—“happiness,” “surprise,” “sadness,” “anger,” “fear,” and “disgust” (e.g., Ekman and Friesen, 1971). As such, negative emotions comprise a broader range of subtypes. Moreover, Unkelbach et al. (2008) have suggested that positive information is more densely clustered in semantic space than negative information, and this leads to processing benefits such as speeded access. As a consequence, a negative mood may only serve to enhance the intrinsic diversity of “negative” as a category and, thus, it may lose its potency as a facilitative agent, in particular, for negative words.

In sum, our study sought to investigate the effect of mood on emotion word recognition, notably by employing strict experimental controls over both the mood-inducing music as well as the word stimuli. Past studies have found mood-congruency effects, but only for the discrete emotions of “happy” and “sad.” We tried to extend these findings to the more general categories of “positive” and “negative.” Our findings did replicate prior studies in terms of the pattern of Emotion × Frequency effects. However, our Mood × Emotion interaction was not driven mood-congruency effects. Instead, it seemed that mood-induced attentional effects differentially modulated responses to emotion words when situated within the context of categories defined only by their valence and arousal.

### Conflict of interest statement

The authors declare that the research was conducted in the absence of any commercial or financial relationships that could be construed as a potential conflict of interest.

## Appendix

**Appendix A:** **Music Stimuli**.

**Music**	**Set**	**Album name (Track No.) or “Song Title” (Artist)**	**Duration (sec)**
**Positive**	**1**	Mozart Essential: Divertimento in D Major, K. 136 (8)	63
		Vivaldi: Concerto in G Major for 2 Mandolins (1)	72^b^
		Made in Dagenham (3)	70^b^
		Pride and Prejudice (14)^a^	69
		“Music Box Dancer” (Frank Mills)	45
	**2**	Oliver Twist (8)^a^	47
		Chocolat (4)	65
		Finding Neverland (23)	66
		Golden Compass (14)	73^b^
		Steel Drum Sounds of the Caribbean (4)	63^b^
	**3**	Dances with Wolves (10)^a^	47
		The Untouchables (6)^a^	68
		Haydn: Flute Concerto in D Major (1)	70^b^
		“Happy Music” (James Last)	60
		The Holidays (5)	54
**Negative**	**1**	“Evil Theme” (Danny Elfman)	58^b^
		The Miraculous Mandarin (1)	74
		Minority Report (3)	61
		Hannibal–Original (1)^a^	57
		Penderecki–Orchestral Works (2)	67^b^
	**2**	Rite of Spring (17)	76^b^
		Lord of the Rings III (28)	72^b^
		War of the Worlds (11)	62^b^
		Iannis Xenakis–Music for Strings (2)	43
		Batman Returns (5)^a^	53
	**3**	“Yeti” (Unknown)	53
		Xenakis: Phlegra/Jalons/Karen/N (2)	62^b^
		Harry Potter and the Chamber of Secrets (18)	53^b^
		Minority Report (5)	76^b^
		Penderecki–Orchestral Works (8)	54

a *Adapted from Eerola and Vuoskoski (2011)*.

b *Looped*.

**Appendix B:** **Word Stimuli**.

**LF**			**HF**		
**Positive**	**Negative**	**Neutral**	**Positive**	**Negative**	**Neutral**
toys	rage	chin	car	war	door
glory	cruel	stiff	strong	death	paper
grin	agony	lamp	music	died	month
intimate	hostile	garment	success	pressure	context
angel	burn	obey	heart	fear	unit
charm	regret	avenue	happy	killed	window
comedy	scared	nursery	leader	failure	method
fame	ugly	spray	cash	pain	plant
liberty	crushed	custom	holiday	broken	teacher
loyal	devil	elbow	truth	crime	chair
sunshine	jealousy	whistle	powerful	accident	machine
bride	slave	invest	travel	fight	museum
circus	misery	hammer	pretty	prison	corner
admired	poison	fabric	freedom	murder	square
trophy	torture	sphere	victory	crisis	manner
nude	jail	lawn	gold	debt	iron
dancer	terror	salad	dinner	afraid	detail
graduate	ambulance	nonsense	excellent	violence	contents
thrill	demon	clumsy	bright	injury	engine
treasure	selfish	umbrella	pleasure	rejected	passage
hug	rat	shy	fun	fat	odd
ecstasy	brutal	coarse	beauty	cancer	author
greet	dread	muddy	beach	guilty	phase
puppy	toxic	basket	treat	victim	metal
cheer	snake	noisy	magic	angry	yellow
awe	rude	lazy	song	hurt	item
merry	vomit	rust	proud	bomb	paint
joyful	insult	insect	wedding	damaged	circle
diamonds	deceive	glacier	exciting	disaster	medicine
heal	slap	bland	gift	gun	bowl
tasty	annoy	vest	kiss	evil	clock
jewels	drown	stove	heaven	crash	shadow
miracle	filthy	golfer	passion	assault	poetry
blossom	spider	violin	humour	destroy	stomach
paradise	suffocate	reserved	laughter	infection	corridor
lust	riot	nun	joke	hate	tool
vacation	despise	mischief	romantic	confused	curious
aroused	trauma	rattle	reward	horror	journal
radiant	anguish	errand	delight	tragedy	gender
witty	scorn	slush	brave	panic	butter

*LF, low frequency; HF, high frequency*.
